# A Digital Microscopic Inspection of Dentinal Defects after Using Endodontic Retreatment Files

**DOI:** 10.1155/2021/6661387

**Published:** 2021-02-05

**Authors:** Ammar AbuMostafa, Hala Almoqayyad, Al-Omari Mohammad

**Affiliations:** Endodontics Division, Riyadh Elm University, Riyadh, Saudi Arabia

## Abstract

**Aim:**

The study aimed at evaluating the incidence of dentinal defects after root canal retreatment with ProTaper Universal retreatment (PTUR) and XP-endo Shaper and Finisher R (XP).

**Materials and Methods:**

Sixty extracted single-rooted human premolars were selected and divided into 4 groups of 15 teeth each. In the negative control group, the teeth were left unprepared. In the positive control group, the teeth were prepared with ProTaper Next and obturated with no further retreatment. In the PTUR and XP groups, the teeth were prepared and obturated followed by removal of the filling material at body temperature using PTUR and XP instruments, respectively. The roots were then sectioned at 3, 6, and 9 mm from the apex and observed under a digital microscope to detect defects.

**Results:**

PTUR group showed significantly higher (*p* value <0.05) incidence of defects than the other groups. Comparison of no defects versus defects between groups in different areas of root canals demonstrated significant difference among the groups in the apical and cervical regions.

**Conclusion:**

Within the limitations of the present study, PTUR files created significantly more dentinal defects than XP files, with most of those defects at the cervical and apical areas of the root canals.

## 1. Introduction

When root canal therapy fails, a nonsurgical approach is often possible [1], which results in a favorable, yet less than ideal outcome to initial treatment [2]. It has been reported that canal preparation and obturation alone can damage root dentin and create fracture [3], making it reasonable to expect that undergoing these procedures twice will increase the incidence of defects. These defects have presented a progressive pattern, wherein preparation alone results in less damage than preparation and filling, which in turn exhibits fewer defects than preparation, filling, and retreatment [4]. The development of new nickel-titanium (NiTi) endodontic instruments is mainly based on changes in design, alloys, and kinematics [5].

Several companies produce NiTi rotary systems for root canal retreatment. Among them is ProTaper Universal retreatment system (Dentsply-Maillefer, Ballaigues, Switzerland) which consists of three files: D1 (tip size 30/taper 0.09, active tip), D2 (tip size 25/taper 0.08, safe-ended), and D3 (tip size 20/taper 0.07, safe-ended) [2]. Another retreatment instrument named D-Race (FKG Dentaire, La Chaux-de-Fonds, Switzerland) was also marketed, which consisted of a sequence of two retreatment files, DR1 (active tip) and DR2 (safe-ended). More recently, XP-endo Finisher R (FKG Dentaire, La Chaux-de-Fonds, Switzerland) was introduced to enhance the removal of gutta percha (GP) in three dimensions. This instrument is made of an alloy called MaxWire, which renders it straight in its martensitic phase at temperatures below 30°C. However, at body temperature, within the root canal, the alloy transforms to its original austenitic phase, rendering it spoon-like at the last 10 mm with a depth of approximately 1.5 mm [6].

Though using NiTi rotary files in cleaning and shaping has several advantages, several microscopic observational studies have demonstrated that instrumentation with these files produced dentinal microcracks [7–9]. Contrary to this, De-Deus et al. [10], through their microcomputed tomography studies, reported that there was no causal relationship between instrumentation with rotary/reciprocating instrumentation systems and microcrack formation.

This warrants the purpose of this study, which is to investigate the incidence of dentinal defects after using two different rotary retreatment systems: ProTaper Universal retreatment (PTUR) and XP-endo Shaper and Finisher R (XP). To the authors' knowledge, there is no published research investigating the association between using XP in retreatment and the incidence of dentinal defect. The null hypothesis stated that there would be no difference in the incidence of dentinal defects of the tested instruments.

## 2. Materials and Methods

This study was assigned an approval number RC/IRB/2019/257, by the Institutional Review Board of the Research Center at Riyadh Elm University (REU), KSA.

### 2.1. Sample Power Calculation

The sample power was calculated using the G power sample power calculator. Using information from previous in vitro studies an effect size of 0.8 for the test groups, a sample size of 15 per group would yield a power of 0.96 for generic *z* tests. Thus, a total of 60 specimens divided among 4 groups were set for the study.

### 2.2. Specimen Selection and Preparation

Sixty human mandibular premolars, extracted for reasons unrelated to this study, were collected from the dental clinics of the Riyadh Elm University, Riyadh, Saudi Arabia, and used in this study after obtaining an approval from the research center.

The inclusion criteria used were as follows: single canal, mature apex, straight root, and not root canal filled. All teeth were visualized under surgical operating microscope (OMS 2350, Zumax, Jiangsu, China) at 25.6x to exclude any roots with visible external cracks.

Teeth were stored in purified filtered water mixed with alcohol solution (Avalon Pharma, Saudi Arabia) and pure glycerin (Sun Care Pharma, Saudi Arabia) at 37°C and stored in an incubator until the time of the study. The crowns were sectioned off by using a low-speed saw (Isomet 11-1180; Buehler Ltd., Evanston, IL) with water cooling, leaving roots approximately 16 mm in length.

Teeth were wrapped in a single layer of aluminum foil (0.016 mm) before embedding them into cylindrical acrylic blocks (12 mm diameter, 2 cm height). After the acrylic set, the foil was removed to create a space that would be filled with silicone material to simulate the periodontal ligament (PDL). The samples were randomly divided into 4 groups, with 15 teeth in each.

In the negative control group, the teeth received no treatment. In the other 3 groups, canal patency was established with size 15 K-file (Dentsply-Maillefer, Ballaigues, Switzerland). Thereafter, canals were prepared with ProTaper Next rotary instruments (Dentsply-Maillefer, Ballaigues, Switzerland) using an X-smart plus motor (XSM, Dentsply-Maillefer, Switzerland). The full sequence of ProTaper Next rotary files (X1, X2, X3, and X4) was used at 300 rpm and torque from 4–5.2 N cm to prepare the canals.

Canals were irrigated with 2 ml of 2% sodium hypochlorite (NaOCl) solution in between each instrument by using a syringe and a 27-gauge needle. The final irrigation was completed using 2 ml 2% NaOCl and 2 ml 17% ethylenediaminetetraacetic acid (EDTA), followed by 2 ml distilled water. All roots were kept moist throughout the experimental procedures.

### 2.3. Canal Filling

Filling of canals was done by a single operator using cold lateral compaction technique following a previously documented protocol [4]. The canals were dried using absorbent paper points (Dentsply-Maillefer, Ballaigues, Switzerland), followed by the introduction of size 40 gutta-percha cones with taper 2% (Dentsply-Maillefer, Ballaigues, Switzerland) as the master cone; the apical 5 mm of which were coated with sealer (AH26 Dentsply-Maillefer, Ballaigues, Switzerland). Accessory medium-fine cones (Dentsply-Maillefer, Ballaigues, Switzerland) were laterally compacted with a size M spreader (Dentsply-Maillefer, Ballaigues, Switzerland) until no further compaction of gutta-percha cones was possible at a depth >5 mm into the root canal. A few teeth were radiographically checked, as a pilot, to ensure the quality of the obturation. All the group samples were stored in distilled water in an incubator at 37°C until the time of sectioning and imaging to allow the sealers to set.

For the positive control group, the teeth were prepared and obturated and not subjected to any retreatment procedures.

### 2.4. Retreatment Procedures

The retreatment procedure was performed by a single operator in a water bath machine (GFL-Germany) filled with water maintained at 37°C. The irrigation solutions were also warmed and maintained at 37°C in a water bath (GFL-Germany).

#### 2.4.1. PTUR Group

Retreatment of the filled canals was first initiated using D1 (size 30, 0.09 taper) to create a reservoir for the solvent material; a drop of Guttasolv (Septodont, France) was introduced into each canal to soften the gutta percha. D2 file (size 25, 0.08 taper) and D3 file (size 20, 0.07 taper) were then used in succession in a brushing motion until the working length was reached. Files were used at a speed of 600 rpm.

#### 2.4.2. XP Group

Retreatment of the filled canals was first initiated using D-Race 1 file at 800 rpm to create a reservoir for the solvent material; a drop of Guttasolv (Septodont, France) was introduced into each canal to soften the gutta percha. Thereafter, the XP-endo Shaper instrument was used at speed 1000 rpm up to the WL and maintained at the WL for 30 seconds using long gentle strokes with amplitude of 3–4 mm. As a finishing stage, the XP-endo Finisher R was used according to the manufacturer's instruction. It was cooled with Endo-Frost (Roeko, Germany) to keep the instrument straight whilst setting the WL measurement. XP-endo Finisher R was inserted in the canal and operated at 800 rpm using in and out motions with amplitude of 7–8 mm for 30 seconds.

In both retreatment groups, the root canals were irrigated using 2% NaOCl between each file and the final irrigation was performed using 2 ml of 2% NaOCl and 2 ml of 17% EDTA, followed by 5 ml of distilled water. Residual root canal filling materials were inspected using loupes with 5x magnification (Ziess, Germany); cycles of instrumentation were repeated till the filling materials are no longer visualized. Periapical radiographs were taken to verify the removal of root filling materials.

### 2.5. Evaluation

Roots were sectioned horizontally at 3, 6, and 9 mm from the apex with a low-speed saw cutting machine (Buehler IsoMet, Japan) with 100 mm diameter and 0.50 mm thick diamond blade under water cooling. The images of the root sections were then photographed using the digital microscope (HIROX KH 7700, Hirox, Tokyo, Japan) at 50x magnification and subsequently assessed for any defects by the authors.

**Figure 1:**
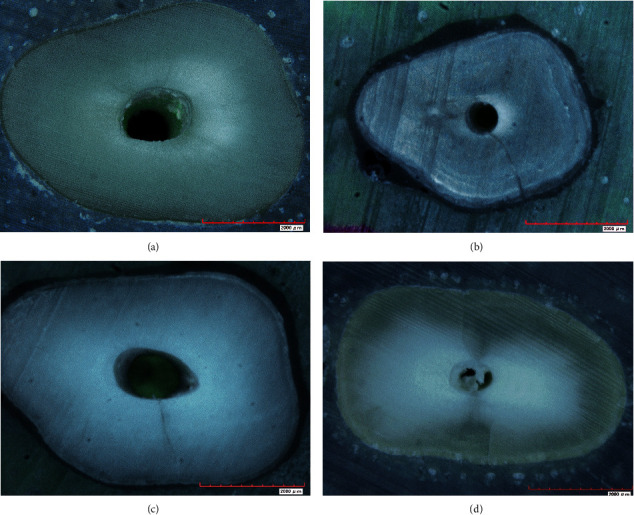
Digital microscope images of the specimen categories. (a) Type A, no defect. (b) Type B, fracture. (c) Type C, partial crack. (d) Type D, craze line.

The specimens were categorized broadly into those that had “no defect” and those with “defect.” The specimens with defects were further categorized into three distinct categories classified according to Shemesh et al. in 2009 (Table 1; Figure 1).

### 2.6. Statistical Tests Used

Descriptive statistics for the type and the presence or absence of defects among the materials were tested separately at each level using the Kruskal–Wallis test. When significant differences were observed, Mann–Whitney *U* tests were used with a Bonferroni correction as a post hoc test. The *p* value for the comparisons was set at *p* < 0.05, Bonferroni correction was applied for the post hoc tests applied for three post hoc comparisons (thus yielding *p* < 0.015 for the post hoc tests). All statistical analyses were performed using the SPSS ver. 25 data processing software (IBM SPSS, Armonk, NY, USA).

## 3. Results

**Figure 2:**
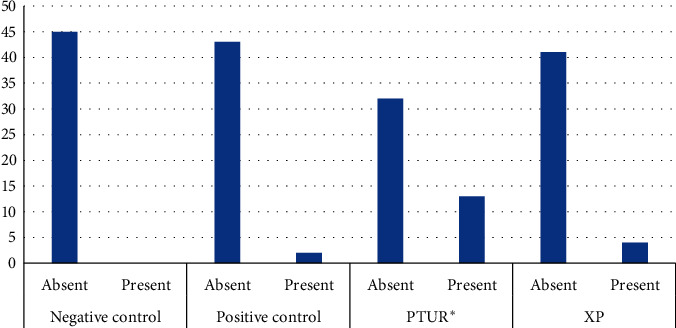
Presence or absence of defects among the groups. ^*∗*^PTUR shows significantly greater presence of defects (Kruskal–Wallis *H* = 23.114, df = 3, *p* < 0.001).

A total of 26 defects were observed among the four groups after root canal retreatment (Table 2). The PTUR group had significantly higher number of overall defects (*n* = 13) when compared to the XP (*n* = 4), positive control (*n* = 2), and negative control (*n* = 0) groups (Figure 2). When the post hoc comparisons were done among the groups, it was observed that PTUR had significantly more defects than XP (Mann–Whitney *U* = 810.00, *p*=0.016). No significant differences were observed between XP and the positive control (Mann–Whitney *U* = 967.5, *p*=0.401) or the negative control (Mann–Whitney *U* = 0.922, *p*=0.042) groups.

Regarding the location of the defects, significant difference among the groups in the apical and cervical regions was found, while there was no significant difference in the middle region (Table 3). The Mann–Whitney *U* test showed that PTUR had significantly more defects than XP in both the apical and cervical regions.

## 4. Discussion

Mandibular premolars were included in the study which were examined under a digital microscope for the presence of any external defects. Studies have reported that the procedure of extraction itself might create such defects [11, 12]. However, the absence of any defect in the negative control group indicates that the teeth were free of cracks; this is consistent with other studies [9, 13, 14]. In the present study, the teeth were sectioned then examined under a digital microscope. Although it can be postulated that the sectioning method might damage the specimens, no defects were found in the specimens of the negative control group.

Over the years, there have been several studies that reported the formation of cracks after NiTi rotary instrumentation with different systems [8, 15, 16]. Beside NiTi files, it has been reported that obturation techniques might cause dentinal defects as well. Blum et al. concluded in their study that the incidence of microcracks increase after obturation [17]. A recent micro-CT-based study also found that intraradicular procedures such as shaping and cleaning and obturation lead to the induction and propagation of dentinal microcracks [18]. In the present study, a gentle compaction was applied by finger spreader to avoid any excessive forces during obturation, and also periodontal ligament simulation was done using a thin layer of silicone material to absorb any force applied during root canal filling procedures and to mimic the clinical condition.

It is also worthy to note that all the retreatment procedures were conducted at body temperature (37°C) to simulate the clinical conditions and to facilitate the phase transformation of the XP files [19].

The incidence of defects was investigated after the retreatment using ProTaper Universal retreatment (PTUR) instruments and XP-endo Shaper and Finisher R (XP). Sectioning and digital microscopic inspection technique were used to detect the presence of defects. It was found that both file systems produced dentinal defects, with a significantly greater incidence (*p* value <0.05) seen with the group of PTUR, thus rejecting the null hypothesis of the study.

The association between the use of NiTi rotary files and dentinal defects is well investigated in the literature. Although most of the reported studies were on initial root canal treatment, there have been a few that evaluated their effects on retreatment procedures, as are presented below.

Different NiTi rotary retreatment files, Mtwo, R-Endo (Micro-Mega, Besancon, France) and D-Race were compared using microscopic assessment. Defects in root dentin were detected in all tested groups without significant difference (*p* > 0.05) [14]. This provided the basis of the null hypothesis of the current study. The present study also reported that the two tested retreatment files created defects; however, there was a statistically significant difference seen.

Micro-CT scanning reports have shown the incidence of dentinal defects to be higher with PTUR files than other tested files [2, 20]. The results of the current study are consistent with theirs, wherein the PTUR files presented with more defects compared to the XP files.

Shemesh et al. [4] used stereomicroscope inspection to investigate the crack formation after using PTUR files in comparison to the use of hand Hedstrom files and found that the retreatment procedures resulted in more defects than initial treatment, while there was no significant difference shown between the groups of retreatments.

Several studies have evaluated the ability of XP-endo Shaper and Finisher R in removing root canal filling [19, 21, 22]; however, no study was found that evaluated its involvement in the formation of dentinal defects after retreatment. Nevertheless, there are studies that evaluated the incidence of dentinal defects of XP-endo Shaper files after primary root canal treatment which reported that no new dentinal microcracks resulted from their usage [5, 23].

The low incidence of defects in the XP group compared to the PTUR group in the present study might be attributed to several factors, among which are the design and geometry. The PTUR instruments (D1, D2, and D3) have various tapers and diameters at the tip, which are size 30, 0.09 taper, size 25, 0.08 taper, and size 20, 0.07 taper, respectively [2]. On the other hand, the central core of XP-endo Shaper is size no. 30 with a 1-degree taper; XP-endo Finisher R has a tip size 30 and is nontapered [6]. The PTUR instruments have a greater taper and stiffness compared to the XP instruments. This also could explain the higher incidence of defects in cervical and apical regions in the PTUR group.

Another factor is the difference in metallurgical properties among the tested instruments. PTUR instruments are essentially made of NiTi in the austenite phase, while XP instruments use a MaxWire technology (Martensite-Austenite electropolish-fleX). The latter provides for better adaption to the root canal anatomy's original shape during preparation and makes the file extremely more flexible [24].

## 5. Conclusions

This study has a few limitations, namely, the sectioning technique used might subject the specimens to additional mechanical stress, the inner preoperative condition of the dentin could not be assessed, and the inability to assess cracks developed along the longitudinal axis of the root.

Under the experimental conditions, all retreatment instruments used in this study created defects in the root dentin. PTUR instruments showed a significantly higher association with creating dentinal defects than XP instruments.

Additionally, when comparing the incidence of defects at the different segments of the root canals, PTUR showed significantly higher incidence of defects in cervical and apical regions, while for XP, there were no significant differences seen.

## Figures and Tables

**Table 1:** Nomenclature and description of specimen categories.

Type	Name	Description
A	No defect	Root dentin devoid of any lines or cracks and where both the external surface of the root and the internal root canal wall had no defects
B	Fracture	A line extending from the root canal space to the outer surface of the root
C	Partial crack	A line extending from the canal walls into the dentin without reaching the outer surface.
D	Craze line	A line extending from the outer surface into the dentin but that did not reach the canal lumen

**Table 2:** Overall distribution of defects^*∗*^.

		Types							
				Type A		Type B		Type C		Type D	
				Count	Column *N* (%)	Count	Column *N* (%)	Count	Column *N* (%)	Count	Column *N* (%)
Group	Negative control			45	28.0	0	0.0	0	0.0	0	0.0
	Positive control			43	26.7	1	16.7	1	8.3	0	0.0
	PTUR			32	19.9	5	83.3	8	66.7	0	0.0
	XP			41	25.5	0	0.0	3	25.0	1	100.0

^*∗*^Only presence of defect is listed in the table; however, both presence and absence are used for the calculation of Kruskal–Wallis *H*.

**Table 3:** Distribution of defects among teeth^*∗*^.

	Group				Kruskal–Wallis *H*	Sig.
	Negative control	Positive control	PTUR	XP		
Apical	0^a^	0^a^	6^b^	1^a^	17.380	0.001^*∗∗*^
Middle	0	2^a^	3^a^	3^a^	3.247	0.355
Cervical	0^a^	0^a^	4^b^	0^a^	12.643	0.005^*∗∗*^
Overall	0^a^	2^a^	13^b^	4^a^	23.114	<0.001^*∗∗*^

^*∗*^Only presence of defect is listed in the table; however, both presence and absence are used for the calculation of Kruskal–Wallis *H*. ^*∗∗*^Differences significant at *p* < 0.01. ^a,b^Differences in superscript indicate significance of difference <0.05 using Mann–Whitney *U* test.

## Data Availability

Data used in this study will be available upon request.
